# Local temperatures predict breeding phenology but do not result in breeding synchrony among a community of resident cavity-nesting birds

**DOI:** 10.1038/s41598-018-20977-y

**Published:** 2018-02-09

**Authors:** Anna Drake, Kathy Martin

**Affiliations:** 1 Department of Forest and Conservation Sciences, University of British Columbia, 2424 Main Mall, Vancouver, British Columbia V6T 1Z4 Canada; 2 Science & Technology Branch, Environment and Climate Change Canada, 5421 Robertson Road, R.R. 1, Delta, British Columbia V4K 3N2 Canada

## Abstract

Weather and ecological factors are known to influence breeding phenology and thus individual fitness. We predicted concordance between weather conditions and annual variation in phenology within a community of eight resident, cavity-nesting bird species over a 17-year period. We show that, although clutch initiation dates for six of our eight species are correlated with local daily maximum temperatures, this common driver does not produce a high degree of breeding synchrony due to species-specific responses to conditions during different periods of the preceding winter or spring. These “critical temperature periods” were positively associated with average lay date for each species, although the interval between critical periods and clutch initiation varied from 4–78 days. The ecological factors we examined (cavity availability and a food pulse) had an additional influence on timing in only one of our eight focal species. Our results have strong implications for understanding heterogeneous wildlife responses to climate change: divergent responses would be expected within communities where species respond to local conditions within different temporal windows, due to differing warming trends between winter and spring. Our system therefore indicates that climate change could alter relative breeding phenology among sympatric species in temperate ecosystems.

## Introduction

In seasonal environments, favorable conditions for producing and rearing young are restricted to a brief temporal window and timing of breeding is a major determinant of individual fitness^1–3^. Photoperiod and temperature are major determinants of such windows within temperate landscapes due to their impact on food abundance and metabolic costs^4–6^. Correlations between local environmental factors, particularly temperature, and clutch initiation are well documented for both migrant and resident bird species^7–10^. Fitness costs associated with mis-timed breeding relative to local conditions are also well documented. For example, individuals that begin nesting too early in a given year may experience adverse weather conditions that result in clutch loss, lower quality eggs, or reduced personal survival or condition^5,11,12^. Individuals breeding too late may miss peak resource availability^13,14^, lay fewer eggs^15–17^, or have inadequate time to produce a second brood or to re-nest if an earlier nest is depredated^18^. Young from late nests additionally have less time to achieve independence before conditions deteriorate, which may result in lower recruitment rates than early-initiated clutches^15,17^.

The role of local ecological factors that are not weather-dependent in altering avian breeding phenology is less studied. Periodic outbreaks in forest insects may remove caloric constraints in early spring and thereby enable females to advance laying dates^19^. In cavity-nesting vertebrate communities, nest site limitation may also delay breeding. For birds that are secondary cavity-nesting species (those relying exclusively on existing cavities for nesting), this may be driven by competition for breeding sites^20,21^; for facultative excavators (species that can make cavities, but will also reuse established sites), limited cavity availability can result in delays associated with cavity excavation^22^. Such ecological factors, combined with environmental cues, should influence timing in communities. Thus annual variation in breeding phenology has strong fitness implications, yet few studies have examined the combined influence of weather factors and ecological resource constraints in driving annual variation within groups of sympatric species.

In this study, we use a 17-year dataset (1995–2011) to compare breeding phenology at the community-level for a group of eight resident, largely insectivorous, tree cavity-nesting bird species in the interior of British Columbia, Canada (four woodpeckers, three small insectivores, and one small owl species (Table 1)). Although dietary differences are likely to influence when these species initiate breeding, we predicted that annual variation in lay date would show similar directionality among our species, such that breeding would occur relatively earlier in some years and relatively later in others, across the community. We test this using pair-wise correlation analyses. We then examine whether timing of breeding is correlated with local weather cues (temperature and/or rainfall) prior to breeding and whether there is an additional effect of local ecological factors (mountain pine beetle abundance (hereafter, MPB) and cavity availability). We predicted that these resident species would show similar responses to local weather prior to breeding. Additionally, we predicted that increased MPB availability for all species (except the owl), and nest site availability among secondary and facultative cavity-nesters, would result in earlier breeding dates.

**Table 1:** Interior of British Columbia cavity-nesting species monitored in the study. Nesting strategies and whether these species consume mountain pine beetle (MPB; *Dendroctonus ponderosae*) during its larval (L) or adult (A) stage are noted.

Species	Nesting strategy	MPB consumer Yes/No (stage)
Black-capped chickadee (*Poecile atricapillus*)	Obligate excavator	Y (L, A)
Downy woodpecker (*Picoides pubescens*)	Obligate excavator	Y (L, A)
Hairy woodpecker (*Picoides villosus*)	Obligate excavator	Y (L, A)
American three-toed woodpecker (*Picoides dorsalis*)	Obligate excavator	Y (L, A)
Pileated woodpecker (*Dryocopus pileatus*)	Obligate excavator	Y (L, A)
Red-breasted nuthatch (*Sitta Canadensis*)	Facultative excavator	Y (L, A)
Mountain chickadee (*Poecile gambeli*)	Secondary cavity-nester	Y (L, A)
Northern saw-whet owl (*Aegolius acadicus*)	Secondary cavity-nester	N

## Results

### Breeding synchrony

**Figure 1:**
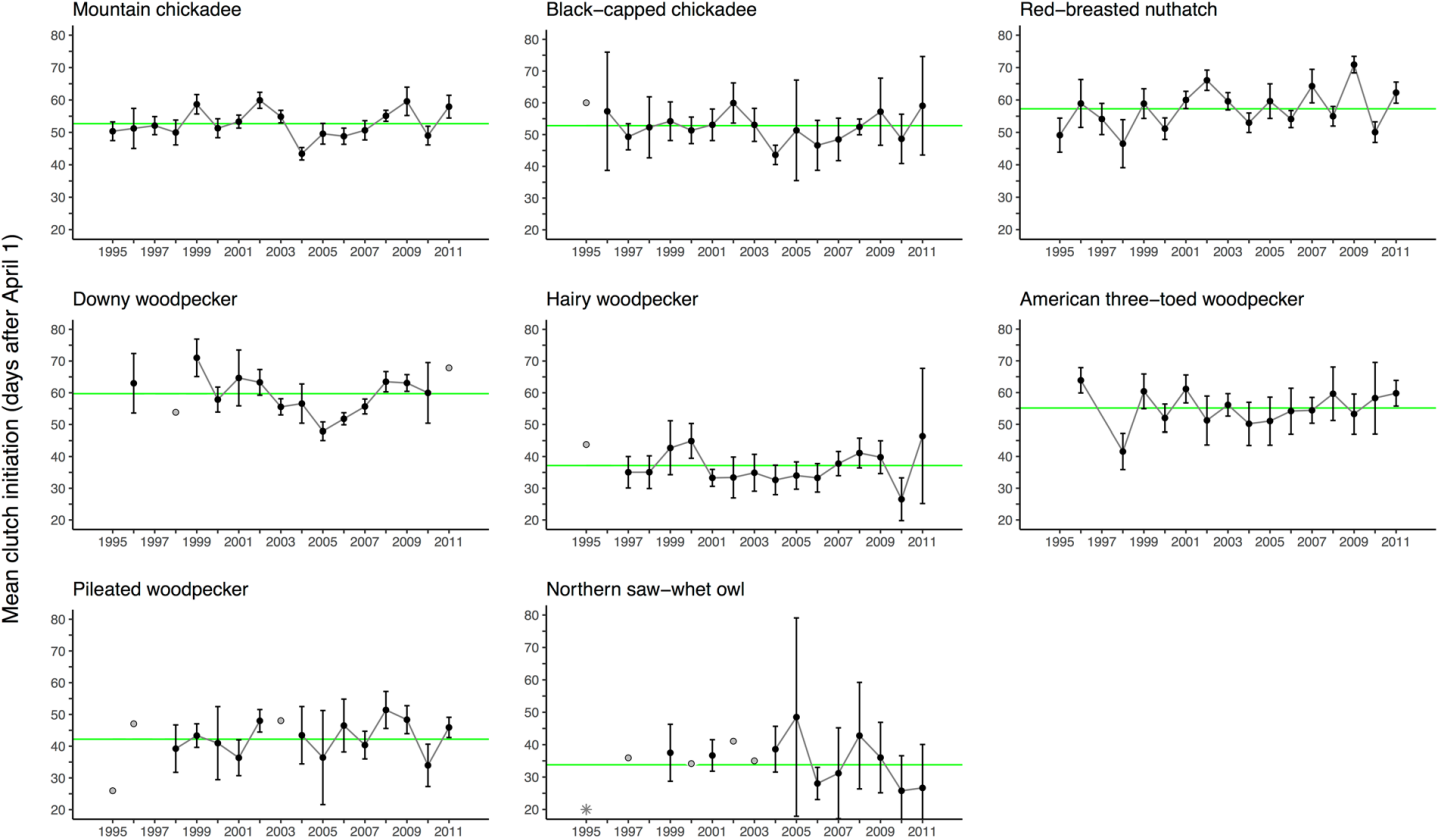
Annual mean clutch initiation (days after April 1 ± 95% CI), for resident cavity-nesting bird species breeding in the interior of British Columbia. The horizontal green line for each species indicates the multi-year average initiation date for that species over the study period. For northern saw-whet owl, (*) indicates one nest in 1995 that was initiated prior to April 1.

Our resident species differed in when, on average, they initiated breeding. Northern saw-whet owl and hairy woodpeckers showed the earliest mean breeding dates (multi-year average: May 4^th^ and May 8^th^, respectively; Fig. 1) while red-breasted nuthatch and downy woodpecker bred the latest (May 28^st^ and May 31^st^, respectively; Fig. 1). All species included in our analysis showed among-year variation in mean clutch initiation date, but only 2 of our 28 possible species dyads showed a significant correlation in timing (Table 2). Northern saw-whet owl, hairy, and pileated woodpecker showed the greatest independence in timing among-years (Table 2). In contrast, the three smallest insectivores showed some correlation in timing of breeding: mountain chickadee timing corresponded with that of black-capped chickadee (Pearson’s r = 0.84, p < 0.001, n = 16 years) and, to a lesser degree, with that of red-breasted nuthatch (Pearson’s r = 0.66, p = 0.05, n = 17 years).

**Table 2:** Pearson’s correlation for mean annual clutch initiation date (n = years) among resident cavity-nesters in the interior of British Columbia. Species whose timing are significantly correlated are denoted ***≤0.001, **≤0.01, *≤0.05. See also Fig. 1.

	Mountain chickadee	Black-capped chickadee	Red-breasted nuthatch	Downy woodpecker	Hairy woodpecker	Pileated woodpecker	American three-toed woodpecker
Black-capped chickadee	0.84*** (16)						
Red-breasted nuthatch	0.66* (17)	0.58 (16)					
Downy woodpecker	0.63 (13)	0.54 (13)	0.21 (13)				
Hairy woodpecker	0.50 (15)	0.45 (15)	0.25 (15)	0.34 (12)			
Pileated woodpecker	0.50 (13)	0.36 (13)	0.38 (13)	0.27 (11)	0.48 (13)		
American three-toed woodpecker	0.30 (15)	0.27 (15)	0.29 (15)	0.59 (13)	0.21 (14)	0.09 (13)	
Northern saw-whet owl	−0.02 (10)	0.00 (10)	0.10 (10)	−0.07 (9)	0.07 (10)	0.05 (10)	−0.29 (10)

### Weather cues

**Figure 2:**
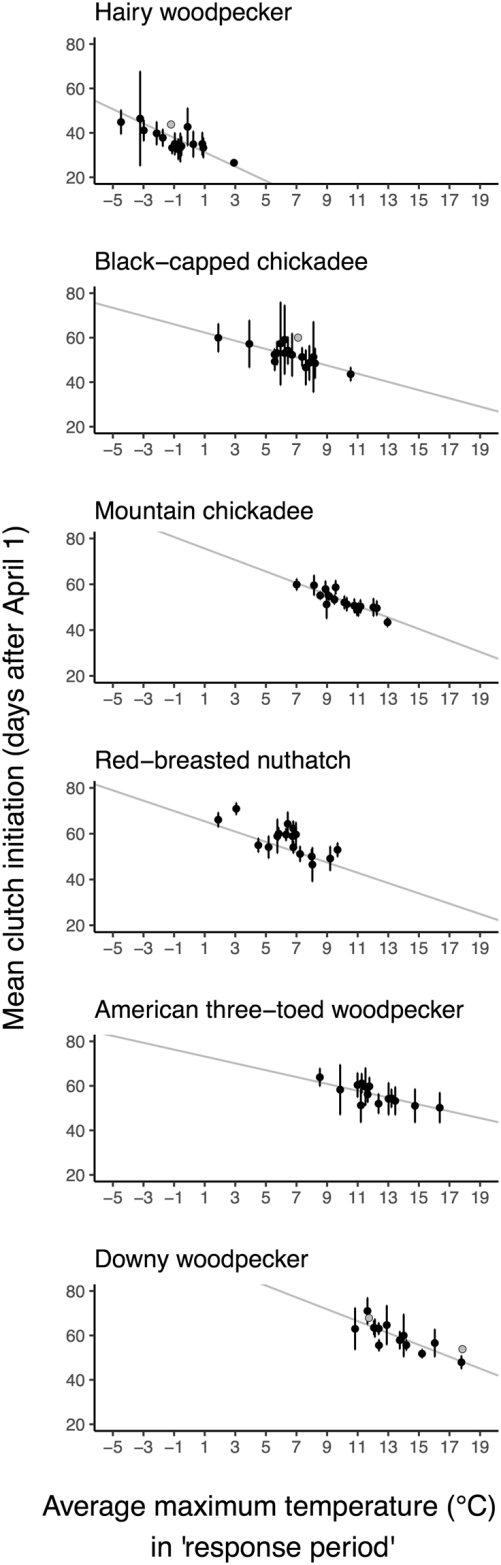
Mean annual clutch initiation dates (days after April 1 ± 95% CI) of cavity-nesting birds in relation to average maximum temperatures in the interior of British Columbia during species-specific response periods (see text and Fig. 3). Species are ordered by their multi-year average clutch initiation date (earliest to latest).

**Figure 3:**
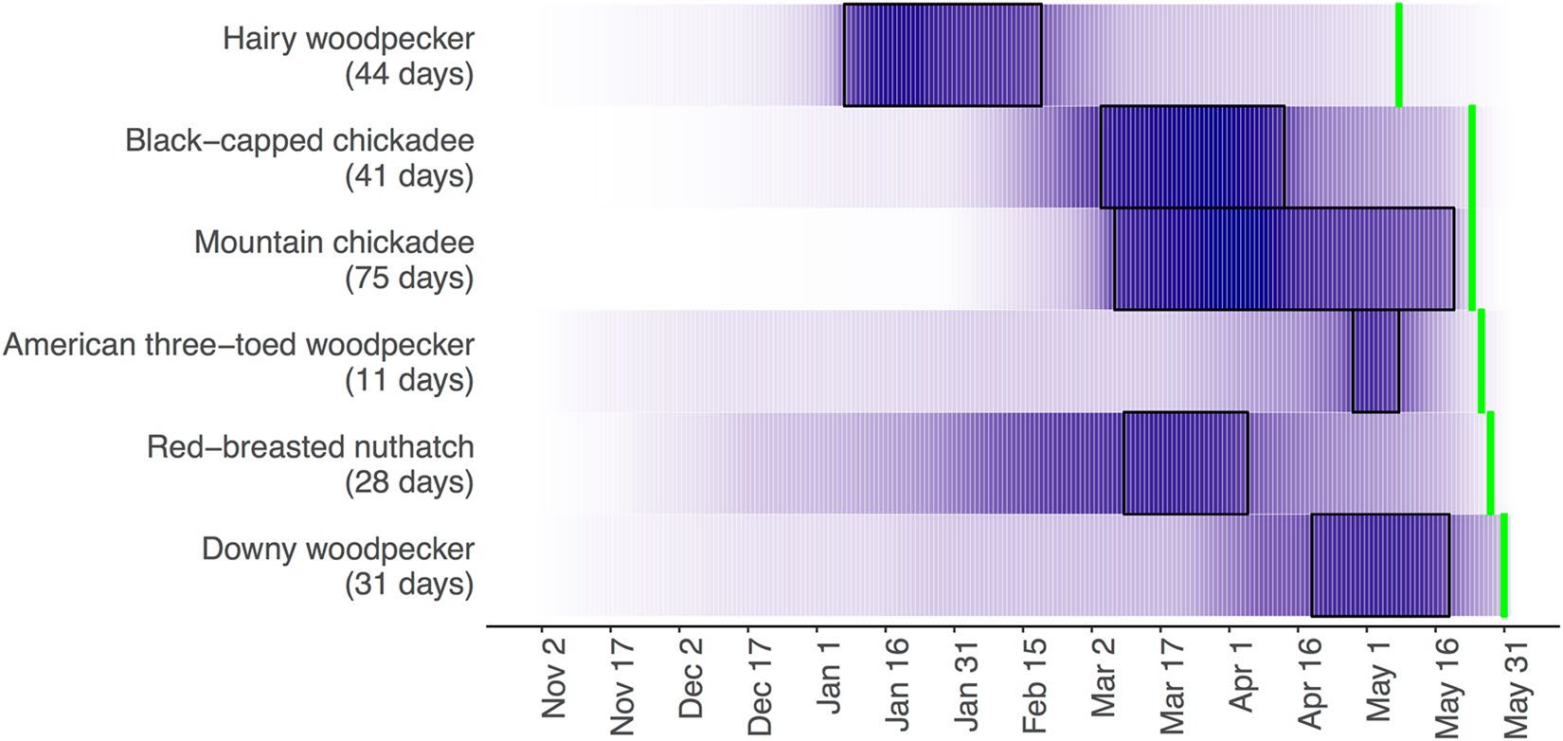
Critical temperature periods correlated with the breeding phenology of resident cavity-nesting bird species breeding in the interior of British Columbia. Model support (AIC weight) for average maximum daily temperatures in predicting clutch initiation dates within different temporal windows prior to breeding is shown in shading. Periods with high support are darkly shaded. Boxed windows represent the best-supported period for each species; the number of days encompassed by this period is shown in brackets. This period is used in all analyses. Vertical green lines indicate the multi-year average clutch initiation date for each species.

The lay dates of the majority of the species we examined (all except pileated woodpecker and northern saw-whet owl) showed a negative relationship (95% CI) with average daily maximum temperatures (i.e. breeding occurred earlier in years that were warmer (Table 3, Fig. 2)). Models using average daily minimum, and average daily mean temperatures were comparable to average daily maximum temperatures for explaining hairy woodpecker phenology. The temporal period in which temperatures best predicted lay dates differed markedly among species, as illustrated in Fig. 3: hairy woodpecker clutch initiation dates corresponded most strongly to temperatures in late winter (maximum temperature best window: Jan 7-Feb 19; minimum and mean temperatures: Jan 14-Jan 24), while higher maximum temperatures in March, April and May were associated with advanced clutch initiation for chickadee species (best windows for black-capped: March 4-April 13, mountain chickadee: March 7-May 20) and red-breasted nuthatch (March 9-April 5). American three-toed woodpecker phenology corresponded with maximum temperatures in late April and early May (April 28-May 8). Phenology of the latest initiator, the downy woodpecker, also most strongly corresponded to maximum temperatures in April and May (April 19-May 19). The interval between these critical temperature periods and clutch initiation was greater than 1 month for black-capped chickadees and red-breasted nuthatch (41 and 53 days respectively) and 2.5 months for hairy woodpecker (78 days; Fig. 3). Mountain chickadee, downy, and American three-toed woodpecker initiated nesting within 1 month of their critical temperature periods (4, 12, and 18 days respectively; Fig. 3).

**Table 3:** The influence of the average maximum temperatures during species-specific ‘response periods’ on the annual timing of clutch initiation for resident cavity-nesting birds in the interior of British Columbia (days after January 1; n = nest records). Red-breasted nuthatch showed an additional effect of cavity availability on timing. Coefficients are the median (95% CI range) values of coefficients derived from 2000 (100 MI × 20 bootstrap) runs of the top-performing model for each species (date ~ fixed effects + a random ‘Year’ effect). The amount of variation explained by model variables is indicated by the marginal R^2^ (variation explained by fixed effects only). Small conditional R^2^ values (the variation explained by fixed effects + the random ‘Year’ effect) indicate large among-individual variation (i.e. low within-species synchrony) in clutch initiation, within-year.

Model Variables	Mountain chickadee (n = 614)	Black-capped chickadee (n = 84)	Red-breasted nuthatch (n = 478)	Downy woodpecker (n = 110)	Hairy woodpecker (n = 108)	Pileated woodpecker (n = 43)	American three-toed woodpecker (n = 85)	Northern saw-whet owl (n = 36)
Intercept	169.13 (164.23, 173.83)	155.17 (149.31, 161.02)	158.68 (154.42, 162.95)	186.81 (176.14, 197.73)	125.53 (123.57, 127.57)	—	165.76 (156.36, 175.82)	—
Maximum temperature	−2.51 (−2.97, −2.03)	−1.85 (−2.66, −1.03)	−2.25 (−2.69, −1.81)	−2.67 (−3.43, −1.90)	−3.23 (−3.45, −1.26)	—	−1.54 (−2.33, −0.83)	—
Cavity availability	—	—	0.01 (0.005, 0.02)	—	—	—	—	—
Marginal R^2^	0.17	0.24	0.18	0.34	0.19	—	0.12	—
Conditional R^2^	0.21	0.34	0.31	0.52	0.30	—	0.22	—

**Figure 4:**
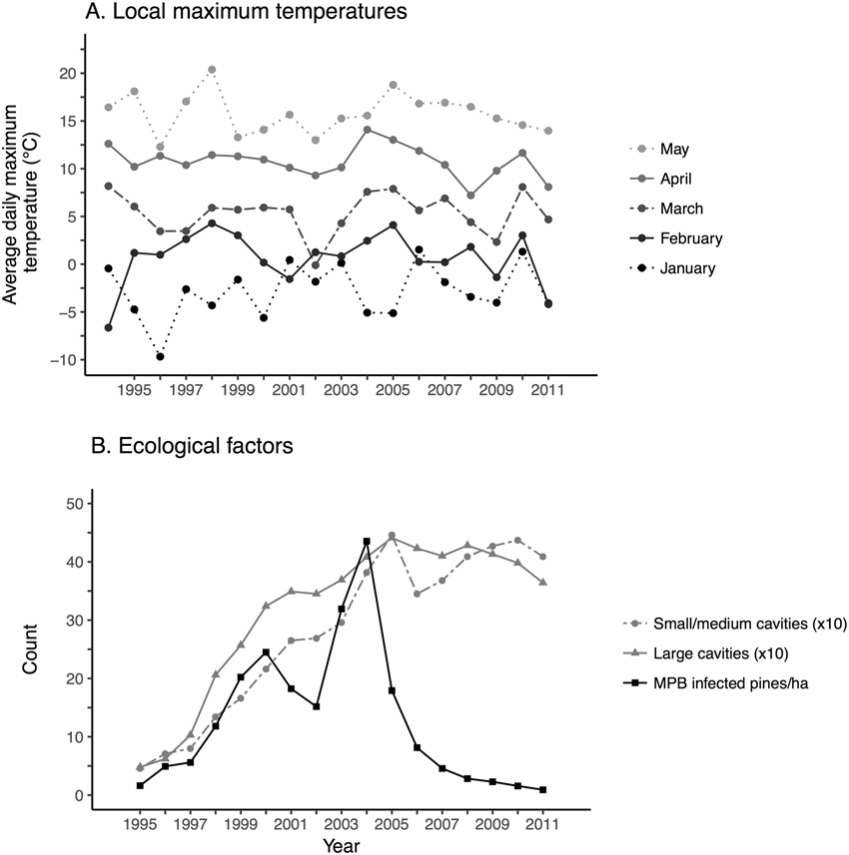
Variation in (**A**) average maximum temperatures and (**B**) ecological factors in the interior of British Columbia over the study period (1995–2011). For ease of presentation, cavity availability has been divided by 10 to allow it to be displayed with MPB abundance. Cavities with entrances <5 cm in diameter were considered small or medium; those with entrances ≥5 cm and <20 cm were considered large.

There was no evidence of a temporal trend between 1995 and 2011 for either temperature (Fig. 4A; p > 0.21 for all months, n = 17 years) or for breeding phenology among seven of the eight cavity-nesters we assessed. Red-breasted nuthatch showed a trend toward later lay dates over the study period (temporal model coefficient (median (95% CI range)): 0.50 days/year (0.26,0.74)). However, this temporal model was not competitive with the daily maximum temperature model reported in Table 3. Local rainfall was not associated with lay dates for any of our species.

### Ecological cues

Contrary to our predictions, we found no support for an additional direct influence of MPB abundance on clutch initiation dates (beyond the possible temperature influences). Similarly, greater cavity availability was not associated with advanced clutch initiation in red-breasted nuthatch, mountain chickadee or northern saw-whet owl. Counter-intuitively, red-breasted nuthatch instead showed weak delays associated with increasing small and medium cavity availability (Table 3).

### Model performance

Marginal and conditional R^2^ values describing the model fit to all nest records are presented in Table 3. To more intuitively present the amount of among-year variation in timing explained by average daily maximum temperatures in our species models, we additionally calculated an R^2^ value using model-predicted versus observed mean annual clutch initiation dates. Temperature variation among years explained 78%, 65%, and 54% of the variation in mean annual clutch initiation dates for American three-toed (n = 15 years), downy (n = 15), and hairy woodpeckers (n = 16), respectively. Temperature explained 77%, 48%, and 48% of the variation in mean mountain chickadee (n = 17 years), black-capped chickadee (n = 17), and red-breasted nuthatch initiation dates among years (n = 17; Fig. 2). None of our candidate explanatory variables described the variance in pileated woodpecker or northern saw-whet owl clutch initiation dates; weather models for both species did not pass our Type 1 error test (see Methods). Small sample sizes (an average of 3.3 recorded nests/assessed year for pileated woodpecker and 3.6 recorded nests/assessed year for northern saw-whet owl) coupled with fewer years of data likely precluded our ability to detect anything but strong relationships for these species.

## Discussion

We examined the underlying influences of weather factors as well as the role of other ecological resource constraints in driving annual variation in breeding phenology over a 17-year period across a suite of sympatric resident bird species within a temperate mixed forest ecosystem. To our knowledge, this is the largest community of sympatrically breeding birds where the drivers of timing of breeding have been examined. As expected, phenology was correlated with temperature within our community. Specifically, local maximum daily temperatures were correlated with clutch initiation dates for 6 of the 8 species we examined (owls and pileated woodpeckers had no detectable response to any weather variables). Contrary to our expectations, however, this commonality did not translate into similar annual variation in clutch initiation among years. Only the small insectivorous species (chickadees and red-breasted nuthatch) showed similar annual variation in clutch initiation. Different annual responses in breeding phenology among species corresponded to different intervals in the time period, prior to breeding, where temperature appeared to be influential. These “critical temperature periods” tended to be positively related to the average onset of clutch initiation for each species: the earliest breeder in our dataset (hairy woodpecker) adjusted clutch initiation in response to winter temperatures (January and February), while the later breeders (the congeneric American three-toed and downy woodpecker) showed the strongest correlations with April and May temperatures. None of our species responded to annual variation in precipitation.

The interval between critical temperature period and average clutch initiation date varied widely among species in our study. Downy and three-toed woodpeckers appeared to respond to local conditions within a period that was close to their average clutch initiation date, as did mountain chickadees. In contrast, black-capped chickadees and red-breasted nuthatch responded to local temperatures more than one month prior to clutch initiation (41 and 53 days, respectively) and hairy woodpeckers appeared to respond to local temperatures more than two months prior to their average lay date (78 days). These lags are longer than is generally reported for birds (less than 1 month^23^) and temperature may therefore be acting as a breeding cue via secondary factors such as insect phenology or abundance^24^.

If distinct critical periods within our community are the product of species-specific food sources that increase in abundance or availability at different threshold temperatures, we might predict a relationship between a species’ response period or average breeding onset and their foraging guild. While our two foliage-gleaning species (mountain and black-capped chickadees) both responded to March temperatures and initiated laying within the same time window, there was no shared response or lay period among the bark-gleaners. Our bark-gleaning congenerics responded to local temperatures ranging from winter (hairy woodpecker) until May (downy and American three-toed woodpecker) and exhibited notably different multi-year average lay dates. The dramatic differences we observed in breeding phenology of *Picoides* woodpeckers, with hairy woodpeckers initiating clutches several weeks earlier than downy woodpeckers (May 8 vs. May 31 respectively) have also been observed in the boreal mixedwood forest of northwestern Quebec where hairy woodpeckers initiated clutches more than three weeks earlier than downy woodpeckers (mean initiation of May 9 vs. May 31, respectively; Pierre Drapeau, *personal communication*). This indicates that our observations may be general among sympatric *Picoides* woodpeckers, although the underlying drivers of such divergence are unknown.

Earlier work in the interior of British Columbia found significant positive relationships between mountain pine beetle numbers and breeding densities for most of the woodpeckers, and both density and fecundity for mountain chickadee and red-breasted nuthatch^22,25–28^. Here, we found no evidence of an additional influence of beetle numbers on the timing of breeding for any of these species. Thus, the observed increased functional and numerical responses reported earlier for our study site do not appear to be related to earlier clutch initiation, but instead to a direct effect of the food subsidy on productivity. Cavity availability also did not appear to influence the breeding phenology of our secondary and facultative tree cavity-nesting species with the exception of a weak, positive relationship for red-breasted nuthatch (0.01 day delay/available cavity; Table 3). Red-breasted nuthatch are facultative excavators and this result may be partly explained by an increase in cavity excavation during the mountain pine beetle outbreak when nuthatch density increased and individuals shifted breeding sites to patches where beetles were abundant but cavities were not^29^.

Our results suggest that the monitoring of a few focal species as a means to understand local responses to climate change is problematic. Warming trends associated with climate change have been heterogeneous across seasons and more pronounced in winter in both Europe and western Canada (e.g.^30,31^). Although we did not detect a temporal trend in average maximum temperatures over our 17-year study period, the interior of British Columbia has experienced an average warming of 1 °C over the past 100 years^32^. This warming has occurred disproportionately in the winter, with an increase of 1.6 °C vs. 0.8 °C in spring and summer combined. We would therefore expect that hairy woodpecker breeding dates have advanced more than other species in our community. That daily maximum temperatures, rather than minimum or means, showed the strongest correlation with phenology in our community is also noteworthy. Within our study region, maxima have increased at a slower rate than minima and, globally, maximum temperatures have increased at approximately half the rate of minimums (1950–2004^33^). Thus resident cavity-nesting species may be somewhat buffered from climate-driven variation influencing their temporal response periods, at least initially. Overall, seasonal differences in temperature increases associated with climate change, combined with different species response periods (as observed in our study), as well as the type of temperature cue used (daily maxima, minima or means) may explain some of the heterogeneous responses of birds to climate change observed in numerous studies (e.g.^34,35^). These differences may therefore not represent a failure by some species to track conditions, but instead species-specific differences in what conditions are being tracked. Further work explicitly quantifying food availability would be needed to establish this. Examining the responses of multiple sympatrically breeding species enables us to refine predictions about the influence of climate change within communities.

## Methods

### Study site and breeding activity

Data on timing of breeding for our cavity-nesting community were collected at Riske Creek and Knife Creek near Williams Lake in south-central British Columbia, Canada (51°52′N, 122°21′W) from 1995–2011. The Riske Creek site is composed of mixed conifer stands with trembling aspen (*Populus tremuloides*) within a grassland-wetland matrix. Knife Creek is largely mixed conifer with some deciduous riparian zones. Further detail about these sites can be found in Martin *et al*.^36^. Thirty-one cavity-nesting bird species are found in the community^36^; we restricted our analyses to the 8 resident species for which we found the highest abundance of nests (Table 1).

Active cavities were identified during systematic nest searches of the field sites that began in early May and ran until the end of July. Nesting attempts were initially identified based on adult behaviour (excavating, carrying food or entering/exiting cavities) or the vocalizations of young^36^. We recorded nest activity periods as the first and last days that a cavity was observed being used (containing nesting material, eggs, or nestlings). Between 1995 and 2004, active cavities within 5.2 m of the ground were accessed using ladders and mirrors to assess the stage of breeding. After 2004, a video camera mounted on a pole (TreeTop Peeper; Sandpiper Technologies, Manteca, CA, USA) was used to identify the stage of breeding in cavities up to 15 m above the ground^37^. The mean difference in cavity height between the period prior to the use of a pole-peeper and the period afterwards was 0.75 m. Some of this shift was due to changing excavator abundance over the monitoring period as higher cavities (>6 m above ground) produced by red-breasted sapsucker and downy woodpecker became more abundant while northern flicker cavities (an average of 3.5 m above ground) decreased in availability^36,38^. We do not consider this shift in mean height to be a source of bias for our phenology data.

Nests were checked, on average, every 3–5 days during their active phase. Clutch initiation and hatch dates were determined for accessible nests using the observed final clutch size and stages of chick development combined with species-specific laying interval, duration of incubation, and nestling period. Nesting attempts found after the young had hatched could not be assigned a clutch initiation date in the field. Similarly, inaccessible cavities were recorded as active based on adult behaviour or begging chicks but could not be assigned a clutch initiation date or hatch date in the field. When not directly observed, fledging was considered to have occurred if chicks were old enough at the last nest check to survive out of the nest and if there was no evidence of predation within or around the cavity. The presence of adults foraging and feeding chicks in the immediate area was also used as an indicator of successful fledging when the event was not observed directly. All fieldwork was carried out in accordance with relevant guidelines and regulations.

### Phenology

Of 1628 nesting attempts for which phenology data were recorded in the field, 352 had clutch initiation date specifically recorded. We used these dates to inform a linear mixed-effects model predicting the timing of clutch initiation for the remaining monitored nesting attempts as follows. Attempts found at the nestling stage were backdated using actual clutch size (where known) or species mean clutch size at the study site along with species-specific lay-rates (eggs/day), plus the mean incubation period from the literature^39^. These dates were then recorded as dates when nests were at the egg stage. Clutch initiation date was then modelled as a function of: the date and stage (pre-lay, egg, or unknown) of the earliest known/backdated nest activity, the latest date the nest was observed active, nest outcome (fledged, failed pre-hatch, failed pre-fledge, or unknown) and the number of visits to the nest by field crews; species identity was included as a random factor (intercept). With our dataset of field-recorded initiation dates (n = 352) we used a 5-fold cross-validation approach with a 20% set-aside to test the predictive capability of the model and to calculate a root mean-squared prediction error (RMSPE) for predicted clutch initiation^40^. The average performance of the model was high (5-run mean: r = 0.97 n = 70.4) and the RMSPE was 3.40 days. Species-specific performance ranged from r = 0.92 to r = 0.98. Our model was then trained using the entire dataset of field-recorded initiation dates (n = 352) and used to predict initiation dates for the remaining nest attempts (n = 1276). Error within predicted dates was incorporated into the final analyses by multiply imputing these values using a Monte Carlo approach^41,42^: predicted dates were adjusted in each imputation using values obtained from random draws of a normal distribution with a mean of zero and a standard deviation of 3.40 days (the RMSPE calculated above).

We subsequently restricted our analyses to first nesting attempts of the season. Known second nesting attempts by the same breeding pair (noted in the field) were ignored and records were additionally limited to the first occupancy of each nest cavity in each year. This removed possible missed re-nests in the same cavity by the same breeding pair as well as late nesting attempts by other species following abandonment or displacement of the first nesting pair. This reduced the final dataset from 1628 to 1570 nesting attempts.

To calculate the mean (±95% CI) annual clutch initiation dates reported in Fig. 1, we imputed 500 datasets, adjusting predicted values using repeated random draws from our normal distribution with a mean of zero and a standard deviation of the RMSPE. We then calculated mean annual initiation dates and standard deviation for each dataset. Reported values are the average of these 500 imputations. We report mean annual clutch initiation date for each species as “early” or “late” relative to the multi-year average for that species (this average is indicated by the horizontal line in Fig. 1).

### Local weather variables

Daily minimum, maximum and mean temperatures (°C) and rainfall data (mm) were obtained from the Environment and Climate Change Canada weather station Williams Lake A (WMO ID 71104; 52°10′48″N, 122°03′00″W; elevation 939.7 m; http://climate.weather.gc.ca). These showed a strong correspondence with incomplete data from a BC Wildfire Service station at the Riske Creek study site (51°57′37″N, 122°30′00″W; elevation 929 m; Station 210) (Pearson’s r = 0.95, and 0.97 for minimum and mean temperature respectively). Monthly average maximum temperatures are shown in Fig. 4A. Precipitation between the two stations also corresponded, albeit less strongly (Pearson’s r = 0.80).

### Ecological variables

Beyond weather, two ecological variables that could impose additional influences on timing of breeding at our field site showed annual variation (Fig. 4B). A mountain pine beetle (MPB) outbreak impacted our study area during the monitoring period, with peak larval availability occurring in 2000, and 2003–2004. This represented a major food pulse year-round for the majority of species in our study and resulted in the increased abundance of resident cavity-nesting species at our field site^26,28,38,43^.

We considered MPB a possible direct driver of phenology (as opposed to the indirect effect of local temperatures on the beetle outbreak) and quantified the availability of this resource as the number of live, MPB infected pines/ha on the study site in each year (for further detail see^38^). The number of pre-existing cavities also changed over the study period. These cavities were used by secondary cavity-nesters and facultative excavators: mountain chickadee, red-breasted nuthatch, and northern saw-whet owl. We quantified cavity availability for these species at the beginning of the breeding period as the number of cavities in each year that were not newly excavated. We tested small and medium-sized cavity (<5 cm diameter) availability for mountain chickadee and nuthatch and large cavity availability (≥5 cm, <20 cm diameter) for the owl: all types of cavities were least numerous in 1995, and most numerous in 2005^38^.

### Analysis

We tested the degree of co-variation in annual mean clutch initiation dates across our community using pair-wise correlation analyses. Comparisons were restricted to years where species had multiple nests monitored and we corrected for multiple comparisons using Benjamini and Hochberg’s^44^ approach to limit false discoveries. Sample sizes varied from 10 years to 17 years (Table 2).

We used a linear mixed-effects modelling approach to describe individual clutch initiation date for each species as a function of our explanatory weather and ecological variables. As above, only years where a species had multiple nests monitored were included. ‘Year’ was included as a random factor to account for the non-independence of data collected within the same year (‘lme4’). Plausible models were competed to determine which of our candidate variables best-explained phenology within our community. To limit the final model candidate set, we used 2-step hierarchical approach to model testing. The initial analyses examined the performance of our weather variables using a sliding window approach (‘climwin’^45^). Average maximum, minimum and mean temperatures and average total daily rainfall were calculated for a moving window between November 1 of the previous year and June 1 of the breeding year. Minimum window size was restricted to 10 days (providing time for a physiological reproductive response to conditions) and the maximum encompassed the entire time period^23,24^. The best time window for each variable was determined using AIC. The predictive performance of all time periods examined, along with the best single window (boxed), is presented in Fig. 3 using AIC model weights.

Given the large number of time widows examined, we calculated the probability that the AIC scores we obtained could have been achieved by chance (i.e. Type I error) by using a response-data randomization program included for this purpose within ‘climwin’. Specifically, we considered our results to be spurious when our model AIC values did not differ significantly (P > 0.05) from those generated from sliding window analyses of 100 randomizations of the response data (for details see^45^). All weather variables that passed the Type I error test and that fell within ΔAICc ≤ 2 of the top performing variable^46^ were subsequently run against a null (random effect of ‘Year’ only) and linear temporal model, models incorporating our ecological variables, and, finally, biologically plausible additive models that included weather and ecological factors together. For model testing, we imputed 100 datasets using repeated random draws (see *Phenology*, above) and bootstrapped each of these (n = 20 per dataset) to obtain our model estimates^42^. Reported coefficients are the medians, and reported 95% confidence intervals are the middle 95% range (or 0.025 and 0.975 quantiles), of these 2000 model runs (Table 3).

The performance of the “best” final model for each species is presented in two ways. First, we used the function sem.model.fits (‘piecewiseSEM’^47^) on models containing ‘Year’ as a random intercept and fixed effects whose beta values did not cross zero within their 95% CI. Marginal and conditional R^2^ values are the median values obtained from 2000 imputation-bootstrap runs, as above (Table 3). To more intuitively present the amount of among-year variation in timing explained by fixed effects in our models we additionally calculated an R^2^ value for predicted versus observed mean annual clutch initiation dates. These values reflect the fit of regression lines presented in Fig. 2. All analyses were run in R (version 3.2.1, R Foundation for Statistical Computing 2015).

### Data availability

All datasets used in this study are readily available from the corresponding author upon request.
